# Supplementary material for: Content-rich biological network constructed by mining PubMed abstracts
Source: BMC Bioinformatics. 2004 Oct 8;5:147. doi: 10.1186/1471-2105-5-147 (PMC528731; doi:10.1186/1471-2105-5-147)
Supplement: Additional File 5 — The original Chilibot query results of the term "long-term potentiation (LTP)" and 22 other terms, limiting the latest references analyzed to the years 1990, 1995, 2000, and 2004. [file 1471-2105-5-147-S5.bz2 › chilibotAdditionalFile5/ltp2000/html/NMDA_ACTININ.html]

 


 **NMDA** and **ACTININ** 
  
Found 12 abstracts in PubMed,  **12 abstracts were retrieved and analyzed**.  


---

 Search Google  |
 PDF files only 
|  EDU domain only 

---

**Interactive relationship** (e.g. stimulation, inhibition, etc)

- alpha  **actinin**  2 in rat striatum localization and interaction with  **NMDA**  glutamate receptor subunits.  Ref: 10925145 Brain Res Mol Brain Res, 2000
- Despite the high expression of this protein in the striatum, only a minority of  **NMDA**  receptors are linked to alpha  **actinin**  2.  Ref: 10925145 Brain Res Mol Brain Res, 2000
- Thus alpha  **actinin**  may play a role in both the localization of  **NMDA**  receptors and their modulation by calcium.  Ref: 9009191 Nature, 1997
- Recently the actin binding protein alpha  **actinin**  2 was shown to bind directly to the  **NMDA**  receptor subunits NR1 and NR2B, suggesting that alpha  **actinin**  2 may function to attach  **NMDA**  receptors to the actin cytoskeleton.  Ref: 9454847 J Neurosci, 1998
- Immunoprecipitation of membrane protein extracts showed that alpha  **actinin**  2 is present in heteromeric complexes of  **NMDA**  subunits, but is not associated with AMPA receptors in the striatum.  Ref: 10925145 Brain Res Mol Brain Res, 2000
- PSD 95 is known to cluster Shaker channels and  **NMDA**  receptors and the latter is known to couple through alpha  **actinin**  2 to the post synaptic cytoskeleton Wyszynski et al.  Ref: 10812072 FEBS Lett, 2000
- This differential regional expression implies that glutamatergic synapses in various parts of the brain differ with respect to their alpha  **actinin**  2 content and thus, potentially, the extent of possible interaction between alpha  **actinin**  2 and the  **NMDA**  receptor.  Ref: 9454847 J Neurosci, 1998
- alpha  **Actinin**  2 binds by its central rod domain to the cytoplasmic tail of both NR1 and NR2B subunits of the  **NMDA**  receptor, and can be immunoprecipitated with  **NMDA**  receptors and PSD 95 from rat brain.  Ref: 9009191 Nature, 1997
- alpha  **Actinin**  2, which binds both actin and  **NMDA**  receptors, dissociated from the receptor clusters, but PSD 95 remained associated with both the synaptic and nonsynaptic receptor clusters, consistent with a proposed cross linking function.  Ref: 9502803 J Neurosci, 1998
- Additional clustering of  **NMDA**  receptors is provided through the binding of NRI subunits to the cytoskeletal protein alpha  **actinin**  2.  Ref: 10443587 Eur J Pharmacol, 1999
- alpha  **Actinin**  2 colocalized with the  **NMDA**  receptor only at spiny synaptic clusters... suggesting a modulatory role in the anchoring of  **NMDA**  receptor at spines.  Ref: 9454832 J Neurosci, 1998
- Thus alpha  **actinin**  2 is appropriately positioned at the ultrastructural level to function as a postsynaptic anchoring protein for  **NMDA**  receptors.  Ref: 9454847 J Neurosci, 1998
- Alpha  **actinin**  alpha  **actinin**  2 is a protein which links the NR1 and NR2B subunits of N methyl D aspartate  **NMDA**  glutamate receptors to the actin cytoskeleton.  Ref: 10925145 Brain Res Mol Brain Res, 2000
- alpha  **Actinin**  2, a member of the spectrin dystrophin family of actin binding proteins, is identified here as a brain postsynaptic density protein that colocalizes in dendritic spines with  **NMDA**  receptors and the putative  **NMDA**  receptor clustering molecule PSD 95.  Ref: 9009191 Nature, 1997
- Interactions of calmodulin and alpha  **actinin**  with the NR1 subunit modulate calcium dependent inactivation of  **NMDA**  receptors.  Ref: 9952395 J Neurosci, 1999

**Parallel relationship** (e.g. studied together, co-existance, homology, etc.)

- the  **NMDA**  receptor interacting proteins alpha  **actinin**  2, PSD 95, and chapsyn.  Ref: 9454832 J Neurosci, 1998
- Differential regional expression and ultrastructural localization of alpha  **actinin**  2, a putative  **NMDA**  receptor anchoring protein, in rat brain.  Ref: 9454847 J Neurosci, 1998
- Because of the importance of  **NMDA**  receptors in modulating the function of the striatum, we have examined the localization of alpha  **actinin**  2 protein and mRNA in striatal neurons, and its biochemical interaction with  **NMDA**  receptor subunits present in the rat striatum.  Ref: 10925145 Brain Res Mol Brain Res, 2000
